# The Social Standing of Dentists

**Published:** 1904-11

**Authors:** 


### Editorial.

### THE SOCIAL STANDING OF DENTISTS.

One of our contemporaries is spending much time, mental effort, and printer's ink in discussing the social side of dentistry. This may be very important to a certain class of mind, but the writer fails to understand why this should disturb the gray matter in any well-considered intellect.

Society, and the world, is made up of a heterogeneous mass of humanity, all the product of evolution, and with an ever-present tendency to a reversion to the intellectual standard of lower forms; in other words, the barbaric element is never entirely eliminated from the human mind, notwithstanding the general uplift through higher civilizing influences. Hence the various ratings we find in the social world. In one class it is ancestors. This has its highest development in the Chinese, where it assumes the garb of a religion. In this country it builds on the "Heralds' College" in England, and in Philadelphia it must bear the stamp of age, and that age must have begun with the cave-dwellers along the banks of the Delaware. In New York City and its environs, not to have been a descendant of the renowned Knickerbockers is not to be worthy of much consideration. Then there is another class who sail with their yachts, run automobiles at the risk of their necks, and try to kill time in a hundred different ways to get even with their income. This class ordinarily requires a clean certificate that the money possessed should have passed through at least two generations to be clean of the smut and soil of labor, the foundation of their millions. This is the barbaric side of society, and its idol is gold and its heaven, if it has any, is paved with gold and precious stones. These sum up its earthly and spiritual desires.

There is another class that regards all social intercourse, worthy the name, to be based on intellectual culture, and the certificate for entrance into this high estate must be clearly defined. Naturally the devotees of this cult are not inclined to mingle with either of the previous classes, neither of which may furnish a clear title to those mansions of a higher, but perhaps narrower, life.

There is still another class, composed, it may be, of many minds entitled to enter the sacred precincts of one or all of the previously named, but prefer to occupy, to them, a broader platform capable of holding all willing and able to aspire to its demands. Its motto is, an intelligent aspiration for the highest. This may be the ambition of the mechanic, the man behind the hoe, the laborer in the trench, the shoemaker on his bench, the man at the anvil, each and all ambitious to become perfected in the world's work.

It is no new cry for some dentists to mourn that they are unable to secure entrance to society, as they understand and appreciate its value. The writer knew one who seemed to feel that he was somehow and in some way deprived of his just rights for the reason that society would not recognize him on account, as he believed, of his occupation.

The world is more and more dividing itself into classes. The lines are quite well defined in the older civilizations, and no one there specially grieves that he is interdicted from passing over the border. This latter phase is as it should be. It is impossible to avoid these separate spheres of activity, or non-activity, for mind naturally falls in with congenial mind, and it is certainly true that these affinities are governed by a natural law controlling the relations of all worlds, physical and spiritual.

The individual who wishes to be other than he is, to receive recognition because of a large bank balance, or to be accepted into the circle of ancestor worship, or in the cultured body that ignores the labor of the hands as unworthy to be compared with that of the brain, lives in a region where the fool dreams.

Dentistry occupies a peculiar position in the social life of the world. It is a mixed profession. It labors with its hands as well as with its brains. It has in the past been largely recruited from the ranks of the semi-educated. The after-experiences have resulted, with many, in an educated body worthy to mingle in any of the refined circles of the world. The number of this class is necessarily limited, but the "curse of labor," if it be a curse, still clings to these as individuals and the body they represent. The set who worship ancestors will have nothing to do with them. The worshippers of the golden idol will thrust them aside. They say, in substance, "The man who cleans teeth and handles instruments is unworthy to sit at my table." This is all very amusing, and yet very natural, but why the man or woman who loves the work that dentistry offers should complain, or feel grieved when debarred entrance to clubs or societies, is an incomprehensible problem to the writer.

That which is needed more than aught else in the dentistry of the period is not recognition by any set of men or women, but a mental poise that aspires to a higher standard of self and professional respect. The great demand of the best thinkers should be that dentists should cultivate a dignified deportment, and aim for the best life possible in our profession, leaving nothing unattempted, however much may be unattained, that will make for the best. There is amply sufficient room in dentistry and in its social life to satisfy the most aspiring mind. Let it form its own clubs, and not wait, hat in hand, while the ballots of the Golden Idol Club reject the applicant. He ought to be rejected. He is trying to enter doors barred against him. It is difficult to see any difference between the man demanding admission to a club and one knocking at the doors of a private mansion insisting that he be placed on the visiting list.

A friend of the writer's in large practice in Europe once said to him, "I never speak to my patients on the street." This was a man who fully recognized his true position. He made no demands, yet no one received greater consideration from all classes, high and low, than he, or was more beloved by all.

The element that makes for social advancement is true self-respect, and with this is correlated character, the last naturally following the first. The man in dentistry is, or should be, a man devoted to science, and science means knowledge. This being developed in the man, he is ever growing towards the highest. Nothing can take this away, death will not destroy it, but over the border it will ever remain the great force, the true wealth of the spirit.

To the writer, therefore, this humiliating aspiration for recognition not only to be in society, so called, but in medical circles, by a certain class of dentists, should be frowned upon as an unworthy thought. It is unmanly and degrading to professional character. Our work is, beyond question, one of the most important to the health and comfort of the race of any of the callings devoted directly, or indirectly, to the aid of humanity. No arguments are here needed to impress this upon dentists, for they all fully appreciate the fact. If the great mission of this profession is thoroughly understood, then live by and for it, and pass by on the other side the sneers of the unthinking. Emphasize this faith by true dignity, and above all remain true to the work to which your life is dedicated, mingling with your own, and your own will never refuse admission into its inner sanctuary. Thus in congenial intercourse the days will pass serenely, and the products of that life will be for the increasing advancement of the profession of your choice, and your own self-respect will grow with years and experience.

